# Bis(1-methyl­piperazine-1,4-diium) tetra­bromidocuprate(II)

**DOI:** 10.1107/S1600536811024184

**Published:** 2011-06-25

**Authors:** Cong-hu Peng

**Affiliations:** a Department of Chemical & Environmental Engineering, Anyang Institute of Technology, Anyang 455000, People’s Republic of China

## Abstract

The title compound, (C_5_H_14_N_2_)[CuBr_4_], was synthesized by hydro­thermal reaction of CuBr_2_ with 1-methyl­piperazine in an HBr/water solution. Both amine N atoms are protonated. The Cu—Br distances in the tetrahedral anion are in the range 2.3809 (11)–2.4131 (11) Å. In the crystal, moderately strong and weak inter­molecular N—H⋯Br hydrogen bonds link the anion and cation units into an infinite two-dimensional network parallel to the *ab* plane.

## Related literature

For related amino coordination compounds, see: Fu *et al.* (2009 ▶); Aminabhavi *et al.* (1986 ▶); Dai & Fu (2008*a* ▶,*b* ▶). For halogen atoms as hydrogen-bond acceptors, see: Brammer *et al.* (2001 ▶). For the chlorine analogue of the title compound, see: Peng (2011 ▶).  
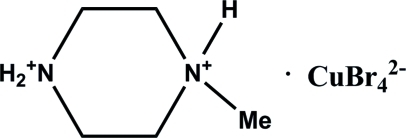


## Experimental

### 

#### Crystal data

(C_5_H_14_N_2_)[CuBr_4_]
*M* *_r_* = 485.36
Orthorhombic, 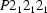

*a* = 9.1933 (18) Å
*b* = 10.341 (2) Å
*c* = 14.255 (3) Å
*V* = 1355.2 (5) Å^3^
*Z* = 4
Mo *K*α radiation
μ = 13.37 mm^−1^
*T* = 298 K
0.20 × 0.05 × 0.05 mm

#### Data collection

Rigaku Mercury2 diffractometer
Absorption correction: multi-scan (*CrystalClear*; Rigaku, 2005 ▶) *T*_min_ = 0.89, *T*_max_ = 1.00
14009 measured reflections
3092 independent reflections
2545 reflections with *I* > 2σ(*I*)
*R*_int_ = 0.079

#### Refinement

*R*[*F* ^2^ > 2σ(*F* ^2^)] = 0.044
*wR*(*F* ^2^) = 0.088
*S* = 1.08
3092 reflections
109 parameters
H-atom parameters constrained
Δρ_max_ = 0.92 e Å^−3^
Δρ_min_ = −0.71 e Å^−3^
Absolute structure: Flack (1983 ▶), 1312 Friedel pairs
Flack parameter: 0.05 (2)

### 

Data collection: *CrystalClear* (Rigaku, 2005 ▶); cell refinement: *CrystalClear*; data reduction: *CrystalClear*; program(s) used to solve structure: *SHELXS97* (Sheldrick, 2008 ▶); program(s) used to refine structure: *SHELXL97* (Sheldrick, 2008 ▶); molecular graphics: *SHELXTL* (Sheldrick, 2008 ▶); software used to prepare material for publication: *SHELXTL*.

## Supplementary Material

Crystal structure: contains datablock(s) I, global. DOI: 10.1107/S1600536811024184/vn2014sup1.cif

Structure factors: contains datablock(s) I. DOI: 10.1107/S1600536811024184/vn2014Isup2.hkl

Additional supplementary materials:  crystallographic information; 3D view; checkCIF report

## Figures and Tables

**Table 1:** Hydrogen-bond geometry (Å, °)

*D*—H⋯*A*	*D*—H	H⋯*A*	*D*⋯*A*	*D*—H⋯*A*
N2—H2*C*⋯Br3^i^	0.90	2.50	3.339 (6)	154
N2—H2*D*⋯Br1^ii^	0.90	2.68	3.354 (5)	133
N2—H2*D*⋯Br2^ii^	0.90	2.76	3.457 (5)	135
N1—H1⋯Br4	0.90	2.55	3.345 (5)	148

Symmetry codes: (i) 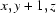; (ii) 
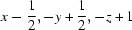
.

**Fig. 1.**
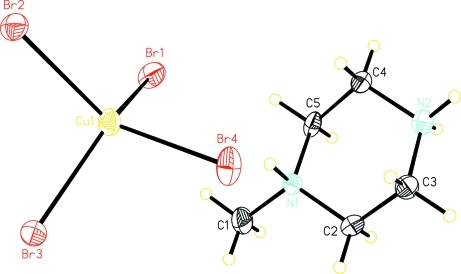
Molecular view of the title compound with the atomic numbering scheme. Displacement ellipsoids are drawn at the 30% probability level.

**Fig. 2.**
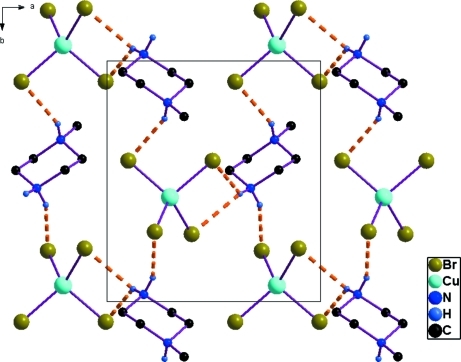
The crystal packing of the title compound viewed along the c axis showing the two-dimensional hydrogen bond network (dashed lines). Hydrogen atoms not involved in hydrogen bonding have been omitted for clarity.

## supplementary crystallographic information

### Comment

Amino derivatives of piperazine have found a wide range of applications in material science, due to their magnetic, fluorescent and dielectric properties. There has also been an increased interest in the preparation of amino coordination compounds (Aminabhavi *et al.* 1986; Dai & Fu 2008*a*; Dai & Fu 2008*b*; Fu, *et al.* 2009). We report here the crystal structure of the title compound, *bis-*(1-methylpiperazine-1,4-diium) tetrabromide copper(II).

The asymmetric unit is composed of one CuBr_4_^2-^ anion and one 1-methylpiperazine-1,4-diium cation (Fig.1). Both amine N atoms are protonated, indicating thus two positive charges on the cation that  balance the two negative charges on the CuBr_4_^2-^ anion. Geometric parameters of the title compound are in the normal range.

In the crystal structure, all H atoms of the amine groups are involved in intermolecular N—H···Br hydrogen bonds with the bond angles ranging from 132.7° to 154.3° and N···Br distances from 3.339 (6)Å to 3.457 (5)Å, respectively. Following the survey by Brammer *et al.*  (2001) the N2—H2D···Br1 and N2—H2D···Br2 H-bonds should be considered to be clearly weaker than the N2—H2C···Br3 and N1—H1···Br4 interactions (Table 1). The hydrogen bonds link the cations and anions into  an infinite two-dimensional network parallel to the *ab*-plane  (Fig.2). The chlorine analogue of the title compound is reported elsewhere in this issue (Peng, 2011).

### Experimental

A mixture of 1-methylpiperazine (0.4 mmol), CuBr_2_ (0.4 mmol) and HBr/distilled water (10ml,1:4) sealed in a teflon-lined stainless steel vessel, was maintained at 100 °C. Blue block -shaped crystals suitable for X-ray analysis were obtained after 3 days.

### Refinement

All H atoms attached to C atoms were fixed geometrically and treated as riding on the parent atoms with C-H = 0.97 Å (methylene) and C-H = 0.96 Å (methyl) with *U*_iso_(H) = 1.2*U*_eq_ (methylene) and *U*_iso_(H) = 1.5*U*_eq_ (methyl). The positional parameters of the H atoms (N1, N2) were initially refined freely, subsequently restrained using a distance of 0.90 Å and in the final refinements treated in riding motion on their parent nitrogen atoms with *U_iso_*(H)=1.2*U_eq_*(N).

### Crystal data

**Table:**

(C_5_H_14_N_2_)[CuBr_4_]	*F*(000) = 908
*M**_r_* = 485.36	*D*_x_ = 2.379 Mg m^−^^3^
Orthorhombic, *P*2_1_2_1_2_1_	Mo *K*α radiation, λ = 0.71073 Å
Hall symbol: P 2ac 2ab	Cell parameters from 3092 reflections
*a* = 9.1933 (18) Å	θ = 3.3–27.5°
*b* = 10.341 (2) Å	µ = 13.37 mm^−^^1^
*c* = 14.255 (3) Å	*T* = 298 K
*V* = 1355.2 (5) Å^3^	Block, blue
*Z* = 4	0.20 × 0.05 × 0.05 mm

### Data collection

**Table:**

Rigaku Mercury2 diffractometer	3092 independent reflections
Radiation source: fine-focus sealed tube	2545 reflections with *I* > 2σ(*I*)
graphite	*R*_int_ = 0.079
Detector resolution: 13.6612 pixels mm^-1^	θ_max_ = 27.5°, θ_min_ = 3.3°
profile data from φ scans	*h* = −11→11
Absorption correction: multi-scan (*CrystalClear*; Rigaku, 2005)	*k* = −13→13
*T*_min_ = 0.89, *T*_max_ = 1.00	*l* = −18→18
14009 measured reflections	

### Refinement

**Table:**

Refinement on *F*^2^	Secondary atom site location: difference Fourier map
Least-squares matrix: full	Hydrogen site location: inferred from neighbouring sites
*R*[*F*^2^ > 2σ(*F*^2^)] = 0.044	H-atom parameters constrained
*wR*(*F*^2^) = 0.088	*w* = 1/[σ^2^(*F*_o_^2^) + (0.0277*P*)^2^] where *P* = (*F*_o_^2^ + 2*F*_c_^2^)/3
*S* = 1.08	(Δ/σ)_max_ < 0.001
3092 reflections	Δρ_max_ = 0.92 e Å^−^^3^
109 parameters	Δρ_min_ = −0.71 e Å^−^^3^
0 restraints	Absolute structure: Flack (1983), 1312 Friedel pairs
Primary atom site location: structure-invariant direct methods	Flack parameter: 0.05 (2)

### Special details

**Table:**

Geometry. All esds (except the esd in the dihedral angle between two l.s. planes) are estimated using the full covariance matrix. The cell esds are taken into account individually in the estimation of esds in distances, angles and torsion angles; correlations between esds in cell parameters are only used when they are defined by crystal symmetry. An approximate (isotropic) treatment of cell esds is used for estimating esds involving l.s. planes.
Refinement. Refinement of F^2^ against ALL reflections. The weighted R-factor wR and goodness of fit S are based on F^2^, conventional R-factors R are based on F, with F set to zero for negative F^2^. The threshold expression of F^2^ > 2sigma(F^2^) is used only for calculating R-factors(gt) etc. and is not relevant to the choice of reflections for refinement. R-factors based on F^2^ are statistically about twice as large as those based on F, and R- factors based on ALL data will be even larger.

### Fractional atomic coordinates and isotropic or equivalent isotropic displacement parameters (Å^2^)

**Table:**

	*x*	*y*	*z*	*U*_iso_*/*U*_eq_	
Br1	0.99472 (8)	0.08523 (7)	0.59527 (5)	0.0496 (2)	
Br2	0.89670 (8)	−0.22010 (7)	0.49622 (5)	0.0430 (2)	
Br3	0.72439 (8)	−0.21019 (7)	0.72661 (5)	0.04138 (19)	
Cu1	0.79551 (9)	−0.06642 (7)	0.60209 (5)	0.0365 (2)	
Br4	0.60073 (8)	0.08567 (7)	0.61127 (6)	0.0501 (2)	
N2	0.6726 (5)	0.5289 (5)	0.5927 (4)	0.0367 (14)	
H2C	0.7150	0.5985	0.6190	0.044*	
H2D	0.6225	0.5519	0.5411	0.044*	
N1	0.7844 (6)	0.3272 (4)	0.7129 (3)	0.0298 (12)	
H1	0.7703	0.2609	0.6731	0.036*	
C4	0.8043 (7)	0.4533 (7)	0.5656 (5)	0.0384 (16)	
H4A	0.8684	0.5069	0.5281	0.046*	
H4B	0.7756	0.3793	0.5281	0.046*	
C5	0.8841 (7)	0.4074 (6)	0.6529 (5)	0.0364 (16)	
H5A	0.9680	0.3564	0.6348	0.044*	
H5B	0.9181	0.4815	0.6884	0.044*	
C2	0.6566 (7)	0.4061 (7)	0.7413 (4)	0.0413 (17)	
H2A	0.6892	0.4790	0.7786	0.050*	
H2B	0.5922	0.3541	0.7798	0.050*	
C3	0.5744 (7)	0.4550 (7)	0.6565 (5)	0.0397 (17)	
H3A	0.5329	0.3824	0.6228	0.048*	
H3B	0.4953	0.5104	0.6769	0.048*	
C1	0.8613 (8)	0.2694 (7)	0.7956 (5)	0.050 (2)	
H1A	0.7941	0.2181	0.8312	0.075*	
H1B	0.8992	0.3374	0.8344	0.075*	
H1C	0.9398	0.2159	0.7741	0.075*	

### Atomic displacement parameters (Å^2^)

**Table:**

	*U*^11^	*U*^22^	*U*^33^	*U*^12^	*U*^13^	*U*^23^
Br1	0.0533 (5)	0.0344 (4)	0.0610 (5)	−0.0140 (4)	0.0190 (4)	−0.0117 (4)
Br2	0.0539 (4)	0.0333 (4)	0.0418 (4)	−0.0101 (4)	0.0066 (3)	−0.0080 (3)
Br3	0.0424 (4)	0.0365 (4)	0.0452 (4)	0.0005 (3)	0.0098 (3)	0.0034 (3)
Cu1	0.0389 (5)	0.0269 (4)	0.0436 (5)	−0.0011 (4)	0.0015 (4)	−0.0011 (4)
Br4	0.0374 (4)	0.0322 (4)	0.0805 (5)	0.0010 (3)	−0.0113 (4)	−0.0026 (4)
N2	0.033 (3)	0.035 (3)	0.041 (3)	0.002 (3)	−0.003 (3)	−0.001 (3)
N1	0.032 (3)	0.024 (3)	0.033 (3)	0.000 (2)	0.000 (2)	−0.002 (2)
C4	0.043 (4)	0.033 (4)	0.040 (4)	0.008 (3)	0.010 (3)	0.002 (3)
C5	0.026 (4)	0.027 (4)	0.057 (4)	0.006 (3)	0.003 (3)	−0.003 (3)
C2	0.038 (4)	0.046 (4)	0.040 (4)	0.009 (3)	0.011 (3)	0.003 (4)
C3	0.025 (4)	0.047 (4)	0.047 (4)	0.004 (3)	0.005 (3)	0.009 (4)
C1	0.053 (5)	0.048 (4)	0.049 (4)	0.015 (4)	−0.007 (3)	0.007 (4)

### Geometric parameters (Å, °)

**Table:**

Br1—Cu1	2.4131 (11)	C4—H4A	0.9700
Br2—Cu1	2.3809 (11)	C4—H4B	0.9700
Br3—Cu1	2.4059 (10)	C5—H5A	0.9700
Cu1—Br4	2.3869 (11)	C5—H5B	0.9700
N2—C3	1.492 (8)	C2—C3	1.513 (9)
N2—C4	1.493 (7)	C2—H2A	0.9700
N2—H2C	0.9000	C2—H2B	0.9700
N2—H2D	0.9000	C3—H3A	0.9700
N1—C2	1.486 (8)	C3—H3B	0.9700
N1—C1	1.499 (8)	C1—H1A	0.9600
N1—C5	1.503 (8)	C1—H1B	0.9600
N1—H1	0.9000	C1—H1C	0.9600
C4—C5	1.520 (9)		
			
Br2—Cu1—Br4	140.15 (4)	N1—C5—C4	110.1 (5)
Br2—Cu1—Br3	99.28 (4)	N1—C5—H5A	109.6
Br4—Cu1—Br3	99.38 (4)	C4—C5—H5A	109.6
Br2—Cu1—Br1	96.41 (4)	N1—C5—H5B	109.6
Br4—Cu1—Br1	98.24 (4)	C4—C5—H5B	109.6
Br3—Cu1—Br1	129.61 (4)	H5A—C5—H5B	108.2
C3—N2—C4	112.3 (5)	N1—C2—C3	111.1 (5)
C3—N2—H2C	114.7	N1—C2—H2A	109.4
C4—N2—H2C	100.1	C3—C2—H2A	109.4
C3—N2—H2D	109.0	N1—C2—H2B	109.4
C4—N2—H2D	109.9	C3—C2—H2B	109.4
H2C—N2—H2D	110.6	H2A—C2—H2B	108.0
C2—N1—C1	112.2 (5)	N2—C3—C2	110.9 (5)
C2—N1—C5	109.5 (5)	N2—C3—H3A	109.5
C1—N1—C5	112.3 (5)	C2—C3—H3A	109.5
C2—N1—H1	118.5	N2—C3—H3B	109.5
C1—N1—H1	105.1	C2—C3—H3B	109.5
C5—N1—H1	98.6	H3A—C3—H3B	108.1
N2—C4—C5	110.1 (5)	N1—C1—H1A	109.5
N2—C4—H4A	109.7	N1—C1—H1B	109.5
C5—C4—H4A	109.7	H1A—C1—H1B	109.5
N2—C4—H4B	109.7	N1—C1—H1C	109.5
C5—C4—H4B	109.7	H1A—C1—H1C	109.5
H4A—C4—H4B	108.2	H1B—C1—H1C	109.5

### Hydrogen-bond geometry (Å, °)

**Table:**

*D*—H···*A*	*D*—H	H···*A*	*D*···*A*	*D*—H···*A*
N2—H2C···Br3^i^	0.90	2.50	3.339 (6)	154
N2—H2D···Br1^ii^	0.90	2.68	3.354 (5)	133
N2—H2D···Br2^ii^	0.90	2.76	3.457 (5)	135
N1—H1···Br4	0.90	2.55	3.345 (5)	148

Symmetry codes: (i) *x*, *y*+1, *z*; (ii) *x*−1/2, −*y*+1/2, −*z*+1.
